# Sparse Aperture InISAR Imaging via Sequential Multiple Sparse Bayesian Learning

**DOI:** 10.3390/s17102295

**Published:** 2017-10-10

**Authors:** Shuanghui Zhang, Yongxiang Liu, Xiang Li

**Affiliations:** School of Electronic Science and Engineering, National University of Defense Technology, Changsha 410073, China; lyx_bible@sina.com (Y.L.); lixiang01@vip.sina.com (X.L.)

**Keywords:** interferometric inverse synthetic aperture radar (InISAR), sequential multiple sparse Bayesian learning (SM-SBL), sparse aperture (SA), multiple measurement vectors (MMV)

## Abstract

Interferometric inverse synthetic aperture radar (InISAR) imaging for sparse-aperture (SA) data is still a challenge, because the similarity and matched degree between ISAR images from different channels are destroyed by the SA data. To deal with this problem, this paper proposes a novel SA–InISAR imaging method, which jointly reconstructs 2-dimensional (2-D) ISAR images from different channels through multiple response sparse Bayesian learning (M-SBL), a modification of sparse Bayesian learning (SBL), to achieve sparse recovery for multiple measurement vectors (MMV). We note that M-SBL suffers a heavy computational burden because it involves large matrix inversion. A computationally efficient M-SBL is proposed, which, proceeding in a sequential manner to avoid the time-consuming large matrix inversion, is denoted as sequential multiple sparse Bayesian learning (SM-SBL). Thereafter, SM-SBL is introduced to InISAR imaging to simultaneously reconstruct the ISAR images from different channels. Numerous experimental results validate that the proposed SM-SBL-based InISAR imaging algorithm performs superiorly against the traditional single-channel sparse-signal recovery (SSR)-based InISAR imaging methods in terms of noise suppression, outlier reduction and 3-dimensional (3-D) geometry estimation.

## 1. Introduction

With the ability to acquire high-resolution radar images of moving targets, inverse synthetic aperture radar (ISAR) has been used in various applications for both civil and military purposes. However, it can only capture the projected 2-dimensional (2-D) characteristics of the target, which largely limits its application. In particular, the advanced automatic target recognition (ATR) technique shows the need for additional features of targets to serve classification. Therefore, interferometric ISAR (InISAR) has been developed to obtain the 3-dimensional (3-D) information of the target. Differently to the ISAR system, which merely utilizes a single channel to transmit and receive signals, the InISAR system generally includes several specially located channels, with one as both a transmitter and receiver, and the rest as receivers only [1]. Then, the 2-D ISAR images from different channels are obtained, and the phase difference between these are extracted to construct the 3-D geometry of the target. Additionally, the 3-D motion parameter of the target can be estimated during InISAR imaging. This paper mainly focuses on InISAR imaging for sparse-aperture (SA) data, which is still a challenge and is of practical significance.

Data are of SA-type when some parts are missing, which can be caused by either deliberate usage of compressive sensing (CS) to reduce the sampling rate, or by accidental effects from noise and jamming. Additionally, a multifunctional radar can also result in SA data, because it alternately transmits narrow- and wide-band signals to track and image the target simultaneously, and unavoidably receives the incomplete, (i.e., SA) wide-band signal. SA data mainly decreases the image resolution by strong side and grating lobes, and improves the difficulty of the translational motion compensation. We note that the ISAR image is usually sparse and contains only a small region of interest with a clear background. ISAR imaging for SA data is generally accomplished via sparse-signal recovery (SSR). Numerous SSR-based ISAR imaging algorithms have been proposed to suppress side and grating lobes for SA data, such as the deterministic model-based orthogonal matching pursuit (OMP) [2,3], and the statistical model-based sparse Bayesian learning (SBL) algorithm [4,5,6]. These algorithms are effective for single-channel 2-D SA-ISAR imaging. For 3-D InISAR imaging, however, their performance is limited, because InISAR imaging generally requires highly similar and well-matched multi-channel ISAR images to extract the interferometric phase of each scatterer with high accuracy. When the signal-to-noise ratio (SNR) of the radar echo is low, the independent process of each channel may induce a mismatch of multi-channel ISAR images, which degenerates the accuracy of the subsequent interferometric process.

Several reported works have focused on InISAR imaging for SA data [7,8,9,10]. In [7,8,9], the ISAR image in each channel is reconstructed separately via traditional SSR methods, which brings no improvement to the matched degree between ISAR images from different channels. In contrary, the algorithm in [10] utilizes the similarity of multi-channels ISAR images to jointly reconstruct them. However, it does not consider the statistical model, so that manual tuning of the regularization parameter is involved, which limits the algorithm’s adaptability. Additionally, it suffers from low computational efficiency, because the real and imaginary part of the ISAR images are reconstructed separately.

We note that it has been verified that the sparse Bayesian method performs superiorly to the $l_{1}$ regularization SSR algorithm in terms of automatic parameter learning and global optimum searching [11]. In this paper, we propose a novel SA-InISAR imaging algorithm within the sparse Bayesian framework. The reconstructions of multi-channel ISAR images are regarded as a problem of sparse reconstruction for the multiple measurement vectors (MMV) [12], and the multiple sparse Bayesian learning (M-SBL) algorithm [13] is applied to reconstruct multi-channel ISAR images, so as to achieve well-matched 2-D images. We note that M-SBL suffers from low computational efficiency because it involves large matrix inversion. Inspired by [14], we propose computationally efficient sequential multiple sparse Bayesian learning (SM-SBL), for which the unknown variables, including the mean, variance matrix and noise variance, are sequentially updated to maximize the marginal likelihood, so as to avoid the time-consuming large matrix inversion. Following this, the proposed SM-SBL is applied to reconstruct multi-channel ISAR images. We note that the SA data and low-SNR condition inevitably induce considerable outliers. Least square (LS) method-based outlier elimination is further proposed. Experimental results based on simulated data validate the robustness and high computational efficiency of the proposed SM-SBL-based SA-InISAR imaging algorithm.

The paper is organized as follows. Section 2 presents the signal model for InISAR imaging, and Section 3 proposes the InISAR imaging algorithm based on SM-SBL. The experimental results are given in Section 4, and the conclusions are drawn in Section 5. The following notations are utilized throughout the paper unless specifically declared. A matrix and vector are denoted with bold letters (e.g., **A**). For a given matrix, **A**, $A_{i\cdot }$, $A_{\cdot j}$, $A_{i,j}$, $A^{-1}$ and $A^{H}$ denote its *i*th row, *j*th column, *(i,j)*th element, inverse and conjugate transposition, respectively.

## 2. Signal Model

**Figure 1:**
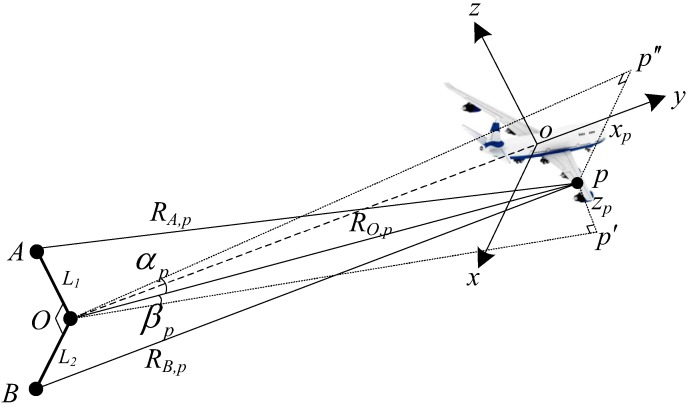
Inverse synthetic aperture radar (ISAR) imaging geometry.

The geometry of the InISAR system is given in Figure 1 and is composed of three vertically positioned antennas, with the antenna *O* as both a transmitter and receiver, and the antennas *A* and *B* as receivers only. $L_{1}$ and $L_{2}$ denote the baselines *OA* and *OB*, respectively. Without loss of generality, we suppose that the target is located in the vertical direction of the plane *AOB*. The coordinate *xoy* is built on the center of gravity of the target, with the axes *ox* and *oz* parallel to *OB* and *OA* respectively, and *oy* along the line of sight (LOS) of the antenna *O*; $p\left(x_{p},y_{p},z_{p}\right)$ is an arbitrary scatterer on the target, whose projections on planes *xoy* and *yoz* are $p^{\prime }$ and $p^{\prime \prime }$, respectively. $R_{O,p}$, $R_{A,p}$ and $R_{B,p}$ denote the instantaneous distances of *p* from three antennas, and $\alpha_{p}$ and $\beta_{p}$ are the azimuth and elevation angle of *p* observed from *O*, respectively. Then, the range-compressed radar echo from *p* is denoted as

(1) $$\begin{array}{cc} S_{O,p}\left(\hat{t},t_{m}\right)= & \sigma_{p}\sin c\left[B\left(\hat{t}-\frac{2R_{O,p}}{c}\right)\right]\cdot \exp\left(-j4\pi f_{c}\frac{R_{O,p}}{c}\right)\\ S_{A,p}\left(\hat{t},t_{m}\right)= & \sigma_{p}\sin c\left[B\left(\hat{t}-\frac{R_{O,p}+R_{A,p}}{c}\right)\right]\cdot \exp\left(-j2\pi f_{c}\frac{R_{O,p}+R_{A,p}}{c}\right)\\ S_{B,p}\left(\hat{t},t_{m}\right)= & \sigma_{p}\sin c\left[B\left(\hat{t}-\frac{R_{O,p}+R_{B,p}}{c}\right)\right]\cdot \exp\left(-j2\pi f_{c}\frac{R_{O,p}+R_{B,p}}{c}\right) \end{array}$$

where $\hat{t}$ and $t_{m}$ denote the fast and slow time, respectively; $f_{c}$, *B* and *c* are the center frequency, bandwidth, and propagation speed of the signal, respectively; and $\sigma_{p}$ represents the reflection coefficient of *p*. It should be noted that the instantaneous distances are related to the slow time $t_{m}$ and are shortened as $R_{O,p}$, $R_{A,p}$ and $R_{B,p}$ for notational simplicity. These instantaneous distances can be derived as follows [10]:

(2) $$\begin{array}{c} R_{O,p}=R_{O,o}+{\tilde{R}}_{p}\\ R_{A,p}\approx R_{O,p}+L_{1}\beta_{p}=R_{O,p}+L_{1}\frac{z_{p}}{R_{O,p}}\\ R_{B,p}\approx R_{O,p}+L_{1}\alpha_{p}=R_{O,p}+L_{2}\frac{x_{p}}{R_{O,p}} \end{array}$$

where $R_{O,o}$ denotes the instantaneous distance between the antenna *O* and the target gravity center *o*, and it also represents the translational motion of the target. ${\tilde{R}}_{p}$ denotes the rotational motion, and can be derived as

(3) $$\begin{array}{cc} {\tilde{R}}_{p}= & {\mathrm{rot}}_{{}^{\theta }}^{y}\left(r_{p}\right)\\ = & x_{p}\cos\left(\omega_{x}t_{m}\right)\sin\left(\omega_{z}t_{m}\right)+y_{p}\cos\left(\omega_{x}t_{m}\right)\cos\left(\omega_{z}t_{m}\right)-z_{p}\sin\left(\omega_{x}t_{m}\right) \end{array}$$

where ${\mathrm{rot}}_{{}^{\theta }}^{y}\left(\cdot \right)$ denotes the obtaining of the *y*-coordinate after the inner vector rotates by an Euler angle of $\theta ={\left[\omega_{x}t_{m}\;\omega_{y}t_{m}\;\omega_{z}t_{m}\right]}^{T}$, and $\omega_{y}$, $\omega_{x}$ and $\omega_{z}$ represent the rotational speeds of the roll, pitch and yaw, respectively; $r_{p}={\left[x_{p}\;y_{p}\;z_{p}\right]}^{T}$ is the location coordinate of *p*. We note that the coherent processing interval (CPI) for ISAR imaging is generally short; the target often only rotates a small angle within the CPI. Therefore, Equation (3) can be approximated with the first Taylor expansion as

(4) $${\tilde{R}}_{p}=x_{p}\omega_{z}t_{m}+y_{p}-z_{p}\omega_{x}t_{m}$$

Substituting Equation (4) into Equation (1), and compensating for the translational motion [15,16] and migration throughout range cell (MTRC) [17], the range profile of *p* is derived as

(5) $$\begin{array}{cc} S_{O,p}\left(\hat{t},t_{m}\right) & =\sigma_{p}\sin c\left[B\left(\hat{t}-\frac{2y_{p}}{c}\right)\right]\cdot \exp\left[-j4\pi \frac{f_{c}}{c}\left(x_{p}\omega_{z}-z_{p}\omega_{x}\right)t_{m}\right]\\ S_{A,p}\left(\hat{t},t_{m}\right) & =\sigma_{p}\sin c\left[B\left(\hat{t}-\frac{2y_{p}}{c}\right)\right]\cdot \exp\left[-j2\pi \frac{f_{c}}{c}\left(2\left(x_{p}\omega_{z}-z_{p}\omega_{x}\right)t_{m}+L_{1}\frac{z_{p}}{R_{o}}\right)\right]\\ S_{B,p}\left(\hat{t},t_{m}\right) & =\sigma_{p}\sin c\left[B\left(\hat{t}-\frac{2y_{p}}{c}\right)\right]\cdot \exp\left[-j2\pi \frac{f_{c}}{c}\left(2\left(x_{p}\omega_{z}-z_{p}\omega_{x}\right)t_{m}+L_{2}\frac{x_{p}}{R_{o}}\right)\right] \end{array}$$

where $S_{i}\left(\hat{t},t_{m}\right),\;i=O,A,B$ denotes the translational compensated range profile from channel *i*. The range differences L1zpzpRoRo and L2xpxpRoRo in the envelope of $S_{A}\left(\hat{t},t_{m}\right)$ and $S_{B}\left(\hat{t},t_{m}\right)$ are eliminated, because they are much shorter than the range resolution cc2B2B. Then, the ISAR image of the scatterer *p* can be achieved by taking the fast Fourier transform (FFT) to the range profiles in Equation (5) with respect to the slow time as

(6) $$\begin{array}{cc} G_{i,p}\left(\hat{t},f\right) & =\sigma_{p}\sin c\left[B\left(\hat{t}-\frac{2y_{p}}{c}\right)\right]\cdot \sin c\left[f-\frac{2f_{c}}{c}\left(x_{p}\omega_{z}-z_{p}\omega_{x}\right)\right]\exp\left(-j\varphi_{i,p}\right)\\ \;\;\;\;\;\;\;\;\;\varphi_{i,p} & =\left\{\begin{array}{cc} 0,\;\;\;\;\;\;\;\;\;\;\;\;\;\;\;\;\;\;\;\; & i=O\\ 2\pi \frac{f_{c}L_{1}z_{p}}{cR_{o}}, & i=A\\ 2\pi \frac{f_{c}L_{2}x_{p}}{cR_{o}}, & i=B \end{array}\right. \end{array}$$

where $G_{i,p}\left(\hat{t},f\right),\;i=O,A,B$ denotes the ISAR image from channel *i*. It is seen that $x_{p}$ and $y_{p}$ are embedded in the phase difference between three ISAR images and can be derived via the interferometric strategy as

(7) $$\begin{array}{c} x_{p}=\frac{cR_{O}\varphi_{B,p}}{2\pi f_{c}L_{2}}\\ z_{p}=\frac{cR_{O}\varphi_{A,p}}{2\pi f_{c}L_{1}} \end{array}$$

It should be noted that the instantaneous azimuth and elevation angle (i.e., $\alpha_{p}$ and $\beta_{p}$ in Figure 1) are time-varying during the CPI and will cause a mismatch of three ISAR images. The time-varying parts of $\alpha_{p}$ and $\beta_{p}$ should be compensated for to match the ISAR images before the process of interferometry, which is also called the ISAR image registration [18].

We now focus on InISAR imaging for SA data. When SSR is utilized to achieve ISAR imaging, the range profiles in Equation (5) and the ISAR images in Equation (6) are regarded as the observation and sparse coefficient, respectively [19]. The range profile in a single range cell can be discretized from Equation (5) as

(8) $$\begin{array}{cc} S_{i}\left(m\right)= & \sum_{p=1}^{P}\sigma_{p}\exp\left[-j4\pi \frac{f_{c}}{c\cdot PRF}\left(x_{p}\omega_{z}-z_{p}\omega_{x}\right)m\right]\cdot \exp\left(-j\varphi_{i,p}\right),\;\;\;\;i=O,A,B \end{array}$$

where *P* denotes the number of scatterers on the target, and the Sinc function in Equation (5) is discarded, because only the main lobe is considered here. Noting that three channels in the InISAR system given in Figure 1 are closely located, the ISAR images they obtain generally share a common sparse pattern, except for the mismatch caused by the time-varying azimuth and elevation angle. Therefore, after compensating for the time-varying part of the azimuth and elevation angle [18], the ISAR images from different channels can be jointly reconstructed by the SSR algorithm for MMV. The range profiles from different channels are modeled in the form of MMV as

(9) $$\begin{array}{cc} S & =\Phi w+n\\ S & =\left[\begin{array}{ccc} S_{O} & S_{A} & S_{B} \end{array}\right]\\ w & =\left[\begin{array}{ccc} w_{O} & w_{A} & w_{B} \end{array}\right] \end{array}$$

where $S\in C^{L\times 3}$, $w\in C^{K\times 3}$ and $n\in C^{K\times 3}$ denote the combined range profile, ISAR image and noise, respectively, and L and K are the number of pulses and reconstructed Doppler cells, respectively. $S_{i}\in C^{L\times 1}$ and $w_{i}\in C^{K\times 1}$, i=O,A,B are the range profile and ISAR image from the *i*th channel, respectively; $\Phi =\left[\phi_{1}\;\phi_{2}\;\cdots \;\phi_{K}\right]$ denotes the partial Fourier matrix, in which ϕk=exp−j2πk2πkMM·I0⋯exp−j2πk2πkMM·IL−1T is the *k*th basis, and *I* is the index sequence of SA. The likelihood of S is assumed to be complex Gaussian distributed with a noise variance of $\sigma^{2}$, as

(10) $$\begin{array}{cc} p\left(\left.S_{\cdot j}\right|w_{\cdot j};\sigma^{2}\right) & =CN\left(\left.S_{\cdot j}\right|w_{\cdot j};\sigma^{2}I\right)\\ & ={\left(\pi \sigma^{2}\right)}^{-L}\exp\left(-\frac{1}{\sigma^{2}}{∥S_{\cdot j}-\Phi w_{\cdot j}∥}_{2}^{2}\right) \end{array}$$

where **I** denotes an identity matrix. To utilize the similarity between ISAR images from different channels, each row of **w** is assigned to be complex distributed with the same variance as

(11) $$p\left(w_{i\cdot };\alpha_{i}\right)=CN\left(\left.w_{i\cdot }\right|0,\alpha_{i}^{-1}I\right)$$

where $\alpha_{i}$ is the reciprocal of the variance or the precision. Then, the full prior of **w** is naturally obtained by combing these row priors as

(12) $$p\left(w;\alpha \right)=\prod_{i=1}^{M}CN\left(\left.w_{i\cdot }\right|0,\alpha_{i}^{-1}I\right)$$

It is seen that the precision vector α controls the sparse degree of rows of **w**. When $\alpha_{i}$ goes to infinity, the corresponding row of **w** is restricted to zero. Differently to the InISAR imaging algorithm based on SBL for single measurement vector (SMV) [20], which models the ISAR image from different channels with a different precision vector, the prior in Equation (12) utilizes a common precision vector to model all ISAR images, which enables them to be reconstructed with a higher matched degree.

## 3. InISAR Based on SM-SBL

### 3.1. SM-SBL

The traditional point estimation algorithms, such as maximum likelihood estimation (MLE) and maximum a posterior (MAP), regard the position of the mode of the likelihood or posterior as the estimation of unknown variables, and they do not require the deriving of a full posterior. In contrary, full Bayesian inference requires the posterior of unknown variables to be derived and estimates them with the mean of posterior, which performs more reliably than the point estimation algorithms, especially for the variable with multi-mode posterior. It has been confirmed in [11] that SBL generally performs better than basis pursuit (BP) and focal underdetermined system solver (FOCUSS) algorithms. Additionally, full Bayesian inference can obtain potentially useful high-order statistical information of unknown variables. In this subsection, the full Bayesian inference-based SM-SBL is derived.

We note that both the likelihood in Equation (10) and the prior in Equation (12) are complex Gaussian distributed. The posterior of **w** is also complex Gaussian distributed as follows [14]:

(13) $$\begin{array}{cc} p\left(\left.w\right|S;\alpha ,\sigma^{2}\right) & =\frac{p\left(\left.S\right|w;\sigma^{2}\right)p\left(w;\alpha \right)}{\int p\left(\left.S\right|w;\sigma^{2}\right)p\left(w;\alpha \right)dw}\\ & =CN\left(\left.w_{\cdot j}\right|\mu_{\cdot j},\Sigma \right) \end{array}$$

with the expectation and covariance matrix given as

(14) $$\begin{array}{cc} \mu & =\left[\mu_{\cdot 1}\;\mu_{\cdot 2}\;\mu_{\cdot 3}\right]\\ & =\sigma^{-2}\Sigma \Phi^{H}S\\ \Sigma & ={\left(A+\sigma^{-2}\Phi^{H}\Phi \right)}^{-1} \end{array}$$

where $A=\mathrm{diag}\left(\alpha_{1},\cdots ,\alpha_{K}\right)$. In SBL, the expectation μ is regarded as the estimation of **w**. However, the process of sparse reconstruction is not yet accomplished, because the hyperparameters in Σ, including α and $\sigma^{-2}$, are still to be learned from the observation. Several strategies have been proposed to deal with hyperparameter learning, such as the type II maximum likelihood algorithm [20], the expectation–maximization (EM) algorithm [21], the variational Bayesian method (VB) [21], and the Markov chain Monte Carlo (MCMC) method [22]. For Gaussian prior and likelihood, these algorithms produce similar results. Herein, we utilize the type II maximum likelihood algorithm to estimate α and $\sigma^{2}$, for which the marginal likelihood is maximized with respect to α and $\sigma^{2}$. The logarithmic likelihood $L\left(\alpha \right)$ is derived as follows [14]:

(15) $$\begin{array}{cc} L\left(\alpha \right) & =\log p\left(\left.S\right|\alpha ,\sigma^{2}\right)\\ & =\log\int p\left(\left.S\right|w;\sigma^{2}\right)p\left(w;\alpha \right)dw\\ & =-3\log\left|C\right|-\sum_{j=1}^{3}S_{\cdot j}^{H}C^{-1}S_{\cdot j}+const \end{array}$$

where *const* denotes the term independent of α and $\sigma^{2}$, and the covariance matrix, **C**, is given by

(16) $$C=\sigma^{2}I+\Phi A^{-1}\Phi^{H}$$

Aiming to maximize $L\left(\alpha ,\sigma^{2}\right)$, the precision vector α and noise variance $\sigma^{2}$ are updated via the EM algorithm [14] as

(17) $$\alpha_{i}^{\left(new\right)}=\frac{1-\alpha_{i}\Sigma_{i,i}}{\frac{1}{3}{∥\mu_{i\cdot }∥}_{2}^{2}}$$

(18) $${\left(\sigma^{2}\right)}^{\left(new\right)}=\frac{\frac{1}{3}{∥S-\Phi \mu ∥}_{2}^{2}}{L-\sum_{i=1}^{K}\left(1-\alpha_{i}\Sigma_{i,i}\right)}$$

where ${\left(\cdot \right)}_{}^{\left(new\right)}$ denote the updated hyperparameter. Then, the M-SBL [13] proceeds by iteratively updating Equations (14), (17) and (18) until convergence is reached and estimates **w** with the newly achieved mean μ. We note that the update of the covariance matrix in Equation (14) involves large matrix inversion. The M-SBL unavoidably suffers from low computational efficiency, and cannot meet the requirement of ISAR image reconstruction, which generally contains several hundreds of samples on both dimensions.

Inspired by [14], we derive SM-SBL to improve computational efficiency, for which the unknown variables are sequentially updated to avoid the time-consuming matrix inversion. To achieve this, the terms related to the *i*th precision, $\alpha_{i}$, are separated from the covariance matrix, **C**, in Equation (16) as

(19) $$\begin{array}{cc} C & =\sigma^{2}I+\alpha_{i}^{-1}\phi_{i}\phi_{i}^{H}+\sum_{m\neq i}^{}\alpha_{m}^{-1}\phi_{m}\phi_{m}^{H}\\ & =C_{-i}+\alpha_{i}^{-1}\phi_{i}\phi_{i}^{H} \end{array}$$

where $C_{-i}$ denotes **C** with the contribution of the *i*-basis vector removed. To derive $L\left(\alpha \right)$, the determinant and inversion of **C** are derived by the matrix determinant and inverse identities as

(20) $$\left|C\right|=\left|C_{-i}\right|\left|1+\alpha_{i}^{-1}\phi_{i}^{H}C_{-i}^{-1}\phi_{i}\right|$$

(21) $$C^{-1}=C_{-i}^{-1}-\frac{C_{-i}^{-1}\phi_{i}\phi_{i}^{H}C_{-i}^{-1}}{\alpha_{i}+\phi_{i}^{H}C_{-i}^{-1}\phi_{i}}$$

Substituting Equations (20) and (21) into Equation (15), we have

(22) $$\begin{array}{cc} L\left(\alpha \right) & =-\frac{1}{2}\left[3\log\left|C_{-i}\right|+\sum_{j=1}^{3}S_{\cdot j}^{H}C_{-i}^{-1}S_{\cdot j}-3\log\alpha_{i}\right.\\ & \;\;\left.+3\log\left(\alpha_{i}+\phi_{i}^{H}C_{-i}^{-1}\phi_{i}\right)-\sum_{j=1}^{3}\frac{{\left|\phi_{i}^{H}C_{-i}^{-1}S_{\cdot j}\right|}^{2}}{\alpha_{i}+\phi_{i}^{H}C_{-i}^{-1}\phi_{i}}\right]+const\\ & =L\left(\alpha_{-i}\right)+\frac{1}{2}\left[3\log\alpha_{i}-3\log\left(\alpha_{i}+s_{i}\right)+\sum_{j=1}^{3}\frac{{\left|q_{i,j}\right|}^{2}}{\alpha_{i}+s_{i}}\right]\\ & =L\left(\alpha_{-i}\right)+l\left(\alpha_{i}\right) \end{array}$$

where

(23) $$L\left(\alpha_{-i}\right)=-\frac{1}{2}\left[3\log\left|C_{-i}\right|+\sum_{j=1}^{3}S_{\cdot j}^{H}C_{-i}^{-1}S_{\cdot j}\right]+const$$

(24) $$l\left(\alpha_{i}\right)=\frac{1}{2}\left[3\log\alpha_{i}-3\log\left(\alpha_{i}+s_{i}\right)+\sum_{j=1}^{3}\frac{{\left|q_{i,j}\right|}^{2}}{\alpha_{i}+s_{i}}\right]$$

(25) $$s_{i}\overset{\Delta }{\;=}\phi_{i}^{H}C_{-i}^{-1}\phi_{i}$$

(26) $$q_{i,j}\overset{\Delta }{\;=}\phi_{i}^{H}C_{-i}^{-1}S_{\cdot j}$$

where $L\left(\alpha_{-i}\right)$ and $l\left(\alpha_{i}\right)$ represent the terms relevant and irrelevant to $\alpha_{i}$, respectively. The fixed-point iteration method is utilized to estimate $\alpha_{i}$. To achieve this, the first derivative of $L\left(\alpha \right)$ with respect to $\alpha_{i}$ is firstly derived as

(27) $$\begin{array}{cc} \frac{\partial L\left(\alpha \right)}{\partial \alpha_{i}}= & \frac{\partial l\left(\alpha_{i}\right)}{\partial \alpha_{i}}\\ = & \frac{1}{2}\left[\frac{3}{\alpha_{i}}-\frac{3}{\alpha_{i}+s_{i}}-\sum_{j=1}^{3}\frac{{\left|q_{i,j}\right|}^{2}}{{\left(\alpha_{i}+s_{i}\right)}^{2}}\right]\\ = & \frac{3\alpha_{i}^{-1}s_{i}^{2}+\left(3s_{i}-\sum_{j=1}^{3}{\left|q_{i,j}\right|}^{2}\right)}{2{\left(s_{i}+\alpha_{i}\right)}^{2}} \end{array}$$

The fixed points of $L\left(\alpha \right)$ are then obtained by setting its first derivative to be zero, including $\alpha_{i}=+\infty$ and

(28) $$\alpha_{i}=\frac{s_{i}^{2}}{\sum_{j=1}^{3}{\left|q_{i,j}\right|}^{2}-3s_{i}}$$

To discuss the features of the two obtained fixed points, the second derivative of $L\left(\alpha \right)$ with respect to $\alpha_{i}$ is further derived as

(29) $$\frac{\partial^{2}l\left(\alpha_{i}\right)}{\partial \alpha_{i}^{2}}=\frac{-3\alpha_{i}^{-2}s_{i}^{2}{\left(s_{i}+\alpha_{i}\right)}^{2}-2\left(s_{i}+\alpha_{i}\right)\left[3\alpha_{i}^{-1}s_{i}^{2}-\left(\sum_{j=1}^{3}{\left|q_{i,j}\right|}^{2}-3s_{i}\right)\right]}{2{\left(s_{i}+\alpha_{i}\right)}^{4}}$$

It is seen that when αi=si2si2∑j=13qi,j2−3si∑j=13qi,j2−3si, the second term of the numerator of ∂2lαi∂2lαi∂αi2∂αi2 is zero. Then we have

(30) $$\frac{\partial^{2}l\left(\alpha_{i}\right)}{\partial \alpha_{i}^{2}}=\frac{-3\alpha_{i}^{-2}s_{i}^{2}{\left(s_{i}+\alpha_{i}\right)}^{2}}{2{\left(s_{i}+\alpha_{i}\right)}^{4}}<0$$

Therefore $L\left(\alpha \right)$ is maximized at αi=si2si2∑j=13qi,j2−3si∑j=13qi,j2−3si. We note that $\sum_{j=1}^{3}{\left|q_{i,j}\right|}^{2}>3s_{i}$ should be satisfied because the precision $\alpha_{i}$ is positive.

When $\alpha_{i}=+\infty$, all the derivatives of $L\left(\alpha \right)$ with respect to $\alpha_{i}$ are zero. Additionally, the sign of the first derivative ${\left.\frac{\partial L\left(\alpha \right)}{\partial \alpha_{i}}\right|}_{\alpha_{i}\to +\infty }$ is decided by the term $3s_{i}-\sum_{j=1}^{3}{\left|q_{i,j}\right|}^{2}$, as given in Equation (27). Therefore, if $\sum_{j=1}^{3}{\left|q_{i,j}\right|}^{2}>3s_{i}$, then ∂Lα∂Lα∂αi∂αiαi→+∞<0, and $L\left(\alpha \right)$ is maximized at αi=si2si2∑j=13qi,j2−3si∑j=13qi,j2−3si and minimized at $\alpha_{i}=+\infty$. If $\sum_{j=1}^{3}{\left|q_{i,j}\right|}^{2}\leq 3s_{i}$, then ∂Lα∂Lα∂αi∂αiαi→+∞>0, and $L\left(\alpha \right)$ is maximized at $\alpha_{i}=+\infty$. Therefore, the maximum points of $L\left(\alpha \right)$ can be summarized as

(31) $$\alpha_{i}=\frac{s_{i}^{2}}{\sum_{j=1}^{3}{\left|q_{i,j}\right|}^{2}-3s_{i}}\;\;\;\;\;\;\;\;\;\;\;\;\;\;\;\;\;\;\;\;\;\;\sum_{j=1}^{3}{\left|q_{i,j}\right|}^{2}>3s_{i}$$

(32) $$\alpha_{i}=+\infty \;\;\;\;\;\;\;\;\;\;\;\;\;\;\;\;\;\;\;\;\;\;\;\;\;\;\;\;\;\;\;\;\;\;\;\;\;\sum_{j=1}^{3}{\left|q_{i,j}\right|}^{2}\leq 3s_{i}$$

These two equations provide a sequential estimation of the precision $\alpha_{i}$. To achieve it, a selection is firstly defined, which includes the basis $\phi_{i}$ whose respective precision is not infinite (i.e., $\alpha_{i}<+\infty$). The number of bases in the selection is dynamic and is adjusted while updating $\alpha_{i}$. For example, if $\phi_{i}$ is included in the selection and $\sum_{j=1}^{3}{\left|q_{i,j}\right|}^{2}\leq 3s_{i}$, then it is eliminated from the selection and $\alpha_{i}=+\infty$. If $\phi_{i}$ is included in the selection and $\sum_{j=1}^{3}{\left|q_{i,j}\right|}^{2}>3s_{i}$, then it is kept and $\alpha_{i}$ is updated with Equation (31). If $\phi_{i}$ is not in the selection and $\sum_{j=1}^{3}{\left|q_{i,j}\right|}^{2}>3s_{i}$, then it is put into the selection and $\alpha_{i}$ is updates with Equation (31). During updating, $s_{i}$ and $q_{i,j}$ can be computed with Equations (25) and (26), respectively, or with the following equations, so as to improve computational efficiency.

(33) $$S_{k}=\phi_{k}^{H}C^{-1}\phi_{k}$$

(34) $$Q_{k,j}=\phi_{k}^{H}C^{-1}S_{\cdot j}$$

Then we have

(35) $$s_{k}=\frac{\alpha_{k}S_{k}}{\alpha_{k}-S_{k}}$$

(36) $$q_{k,j}=\frac{\alpha_{k}Q_{k,j}}{\alpha_{k}-S_{k}}$$

If $\alpha_{k}=+\infty$, then $s_{k}=S_{k}$ and $q_{k,j}=Q_{k,j}$. The Woodbury matrix identity can be utilized to solve Equations (33) and (34) as follows, so as to further reduce computational burden.

(37) $$S_{k}=\sigma^{-2}\phi_{k}^{H}\phi_{k}-\sigma^{-4}\phi_{k}^{H}\Phi \Sigma \Phi^{H}\phi_{k}$$

(38) $$Q_{k,j}=\sigma^{-2}\phi_{k}^{H}S_{\cdot j}-\sigma^{-4}\phi_{k}^{H}\Phi \Sigma \Phi^{H}S_{\cdot j}$$

### 3.2. SM-SBL-Based ISAR Imaging

In this subsection, the proposed SM-SBL is utilized to reconstruct ISAR images from different channels simultaneously. The procedure of multi-channel ISAR imaging based on SM-SBL is given as follows:

(1) Initialize the noise variance by $\sigma_{}^{-2}=0.1\mathrm{var}\left[S\right]$, for which the variance of the combined range profile S is used because the noise variance is approximately similar to it.
(2) Randomly choose a basis $\phi_{i}$ as the initial selection; compute the respective precision $\alpha_{i}$ by Equation (31), that is,
  (39) αi=3ϕi2∑j=13ϕiHS·j2∑j=13ϕiHS·j2ϕi2ϕi2−σ2
  and set $\alpha_{k}=+\infty$ for k≠i.
(3) Update the expectation μ and the covariance matrix Σ with Equation (14); these are scalars when the selection includes only one basis.
(4) Update $s_{k}$ and $q_{k,j}$ with Equations (35) and (36), respectively, for all the bases, including those that are within the selection and those that are not.
(5) Randomly choose a basis from all the bases, and compute $\theta_{i}=\sum_{j=1}^{3}{\left|q_{i,j}\right|}^{2}-3s_{i}$.
(6) If $\theta_{i}>0$ and $\phi_{i}$ is included in the selection, then update $\alpha_{i}$.
(7) If $\theta_{i}>0$ and $\phi_{i}$ is not in the selection, then put it in and update $\alpha_{i}$.
(8) If $\theta_{i}\leq 0$ and $\phi_{i}$ is in the selection, then eliminate it from the selection and let $\alpha_{i}=+\infty$.
(9) Update the noise variance by Equation (18).
(10) Terminate the iteration if convergence is reached, or jump to step (3).

In step (2), the basis used to initialize the selection can be chosen by maximizing the projection of the observation to bases as ϕi=argmaxϕk∑j=13ϕkHS·j2∑j=13ϕkHS·j2ϕk2ϕk2. In step (5), a basis is chosen from the whole bases randomly, or, it can also be chosen as the basis that maximizes the likelihood, so as to improve the convergence rate. Noting that the bases in the selection are much less than those in the original dictionary, Φ, the proposed SM-SBL is much more computationally efficient than M-SBL. Additionally, the update of μ, Σ, $s_{k}$ and $q_{k,j}$ in steps (3) and (4) can be implemented in a sequential manner to further improve computational efficiency. They are sequentially updated in three different cases, which are related to steps (6), (7) and (8), respectively. In step (6), the selection remains unchanged, and only $\alpha_{i}$ is updated. In this case, μ, Σ, $s_{k}$ and $q_{k,j}$ can be sequentially updated as

(40) $$\Sigma^{\left(new\right)}=\Sigma -\kappa_{i}\Sigma_{\cdot i}\Sigma_{\cdot i}^{H}$$

(41) $$\mu^{\left(new\right)}=\mu -\kappa_{i}\Sigma_{\cdot i}\mu_{i\cdot }$$

(42) $$S_{k}^{\left(new\right)}=S_{k}+\kappa_{i}{\left|\sigma^{-2}\Sigma_{\cdot i}^{H}\Phi^{H}\phi_{k}\right|}^{2}$$

(43) $$Q_{k\cdot }^{\left(new\right)}=Q_{k\cdot }^{}+\kappa_{i}\left(\sigma^{-2}\phi_{k}^{H}\Phi \Sigma_{\cdot i}\right)\mu_{i\cdot }$$

where $\kappa_{i}\overset{\Delta }{\;=}{\left[\Sigma_{i,i}+{\left(\alpha_{i}^{\left(new\right)}-\alpha_{i}\right)}^{-1}\right]}^{-1}$. In step (7), $\phi_{i}$ is put into the selection, and $\alpha_{i}$ is updated. In this case, the updates of μ, Σ, $s_{k}$ and $q_{k,j}$ are derived as

(44) $$\Sigma^{\left(new\right)}=\left[\begin{array}{cc} \Sigma +\sigma^{-4}\Sigma_{i,i}\Sigma \Phi^{H}\phi_{i}\phi_{i}^{H}\Phi \Sigma & -\sigma^{-2}\Sigma_{i,i}\Sigma \Phi^{H}\phi_{i}\\ -\sigma^{-2}\Sigma_{i,i}{\left(\Sigma \Phi^{H}\phi_{i}\right)}^{H} & \Sigma_{i,i} \end{array}\right]$$

(45) $$\mu^{\left(new\right)}=\left[\begin{array}{c} \mu -\sigma^{-2}\Sigma \Phi^{H}\phi_{i}\mu_{i\cdot }\\ \mu_{i\cdot } \end{array}\right]$$

(46) $$S_{k}^{\left(new\right)}=S_{k}-\sigma^{-4}\Sigma_{i,i}{\left|\phi_{k}^{H}e_{i}\right|}^{2}$$

(47) $$Q_{k\cdot }^{\left(new\right)}=Q_{k\cdot }-\sigma^{-2}\phi_{k}^{H}e_{i}\mu_{i\cdot }$$

where $\Sigma_{i,i}={\left(\alpha_{i}+S_{i}\right)}^{-1}$, $\mu_{i\cdot }=\Sigma_{i,i}Q_{i\cdot }$ and $e_{i}=\phi_{i}-\sigma^{-2}\Phi \Sigma \Phi^{H}\phi_{i}$. In step (8), $\phi_{i}$ is eliminated from the selection, and $\alpha_{i}$ is set as $\alpha_{i}=+\infty$. In this case, μ, Σ, $s_{k}$ and $q_{k,j}$ can be sequentially updated as

(48) $$\Sigma^{\left(new\right)}=\Sigma -\frac{1}{\Sigma_{i,i}}\Sigma_{\cdot i}\Sigma_{\cdot i}^{H}$$

(49) $$\mu^{\left(new\right)}=\mu -\frac{1}{\Sigma_{i,i}}\Sigma_{\cdot i}\mu_{i\cdot }$$

(50) $$S_{k}^{\left(new\right)}=S_{k}+\frac{1}{\Sigma_{i,i}}{\left|\sigma^{-2}\Sigma_{\cdot i}^{H}\Phi^{H}\phi_{k}\right|}^{2}$$

(51) $$Q_{k\cdot }^{\left(new\right)}=Q_{k\cdot }^{}+\frac{1}{\Sigma_{i,i}}\left(\sigma^{-2}\phi_{k}^{H}\Phi \Sigma_{\cdot i}\right)\mu_{i\cdot }$$

The renewed expectation and covariance matrix can be achieved by deleting the *i*th row of $\mu^{\left(new\right)}$ and the *i*th row and column of $\mu^{\left(new\right)}$.

### 3.3. Outlier Elimination and 3-D Rotational Rate Estimation Based on LM

The SM-SBL-based ISAR imaging method proposed in the former subsection can simultaneously produce three ISAR images from three different channels. The ISAR images from channels *A* and *B* are then conjugately multiplied by that from channel *O* to extract the interferometric phase $\varphi_{i,p}$ in Equation (6), which can be further utilized to estimate the 3-D coordinate of each scatterer by Equation (7). We note that the SA data decreases the quality of ISAR images and inevitably induces outliers during estimation of the 3-D coordinate of each scatterer. In this subsection, an outlier elimination method based on LM is proposed. Additionally, the 3-D rotational rate of the target is estimated during outlier elimination.

The Doppler of each scatterer on the ISAR image is given in Equation (6) as

(52) $$f_{p}=-\frac{2f_{c}}{c}\left(x_{p}\omega_{z}-z_{p}\omega_{x}\right)+\Delta f,\;\;\;p=1,\cdots ,P$$

where Δf and *P* represent the Doppler error and number of scatterers, respectively. Equation (52) can be rewritten as

(53) f=Aω

where $f=\left[\begin{array}{c} f_{1}\\ \vdots \\ f_{P} \end{array}\right]$, $A=\left[\begin{array}{ccc} \frac{2f_{c}}{c}x_{1} & -\frac{2f_{c}}{c}z_{1} & 1\\ \vdots & \vdots & \vdots \\ \frac{2f_{c}}{c}x_{P} & -\frac{2f_{c}}{c}z_{P} & 1 \end{array}\right]$ and $\omega =\left[\begin{array}{c} \omega_{z}\\ \omega_{x}\\ \Delta f \end{array}\right]$. Therefore, the LSM can be utilized to estimate the 3-D rotational rate of target as

(54) $$\hat{\omega }={\left(A^{T}A\right)}^{-1}A^{T}f$$

Then, the *x* and *z* coordinates of the rotational rate of the target can be estimated as ${\hat{\omega }}_{x}=\hat{\omega }\left(2\right)$ and ${\hat{\omega }}_{z}=\hat{\omega }\left(1\right)$, respectively. Subsequently, the outliers can be detected by

(55) $$\left|f_{p}-\left(\frac{2f_{c}}{c}x_{P}{\hat{\omega }}_{z}-\frac{2f_{c}}{c}z_{P}{\hat{\omega }}_{x}\right)\right|>\delta$$

It is seen that a scatterer is regarded as an outlier when its Doppler error exceeds the preset threshold δ, which is suggested to be set as 0.1–0.5 Hz. The smaller the value it is set at, the more outliers that are detected. The motion estimation and outlier elimination in Equations (54) and (55) can be iterated to improve the estimate accuracy, and the threshold δ is kept decreased during the iteration. Generally, two to three iterations are enough to achieve satisfactory accuracy.

**Figure 2:**
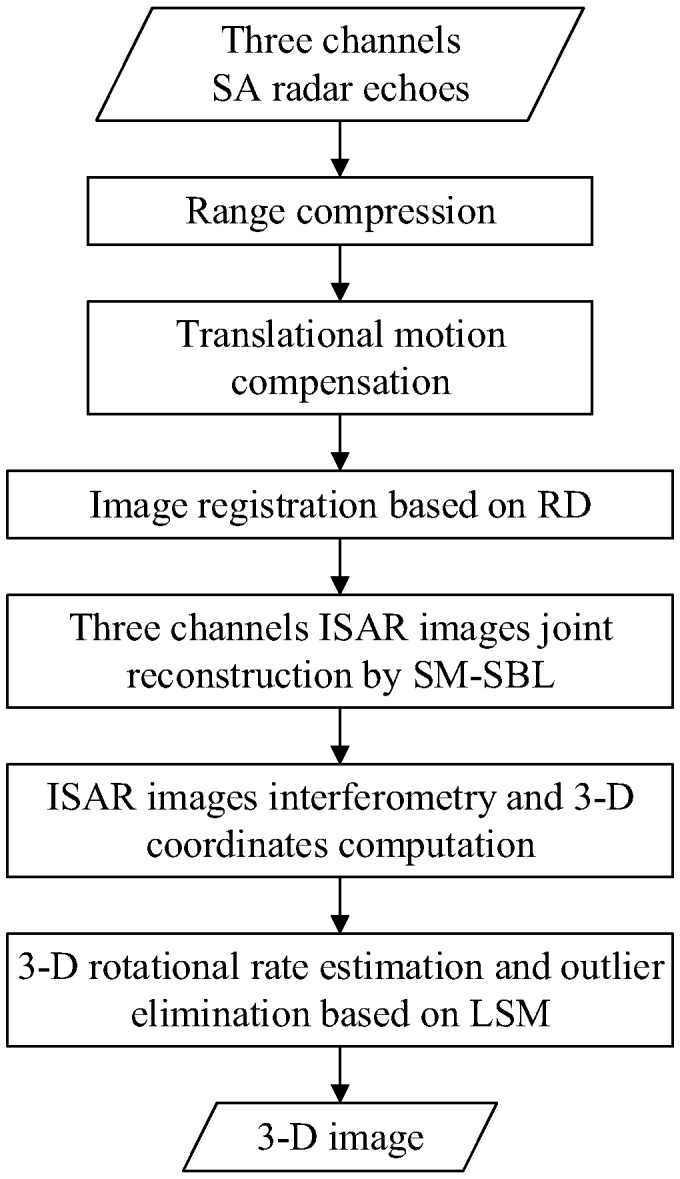
Flowchart of sparse-aperture interferometric inverse synthetic aperture radar (SA-InISAR) imaging sequential multiple sparse Bayesian learning (SM-SBL).

The flowchart of the proposed SM-SBL-based SA-InISAR imaging method is given in Figure 2. Firstly, the range compression and translational motion compensation are implemented to the three channels’ SA radar echoes to obtain the three channels’ range profiles. Then, the traditional range-Doppler (RD) imaging method is utilized to achieve image registration [18]. Subsequently, the three channels’ ISAR images are jointly reconstructed by SM-SBL. The final 3-D image of the target can then be obtained through the processes of ISAR image interferometry, 3-D coordinate derivation, 3-D rotational rate estimation and outlier elimination, successively.

## 4. Experiments

**Figure 3:**
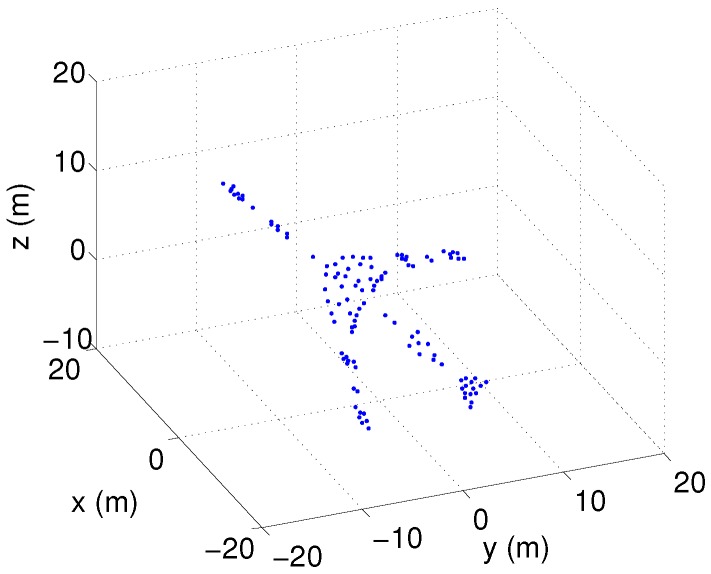
3-Dimensional (3-D) scatterer model of airplane.

This section utilizes a set of simulated data of an airplane to validate the performance of the proposed SM-SBL-based InISAR imaging method. As shown in Figure 3, the simulated airplane contains 113 scatterers. The InISAR system is composed of three channels, which are located as shown in Figure 1. The baselines $L_{1}$ and $L_{2}$ are set as 2 m. The initial range and rotational speed of the target are set as 20 km and (0.01, 0, 0.02) rad/s, respectively. The radar is assumed to transmit a linear frequency modulation (LFM) signal whose central frequency, bandwidth, pulse width and pulse repetition frequency (PRF) are 9 GHz, 600 MHz, 100 μs and 100 Hz, respectively. The test data contained 256 pulses, and each pulse contained 256 samples. Complex Gaussian noise was added to each pulse to simulate the noise environment, which made the SNR of the radar echo fall to 5 dB.

**Figure 4:**
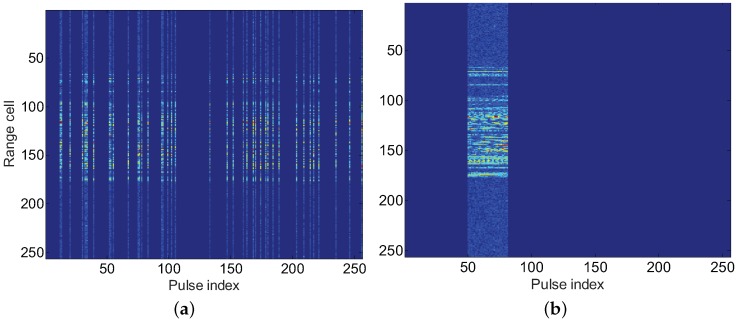
Range profile sequence for two kinds of sparse-aperture (SA) data. (**a**) RMS; (**b**) GMS.

We extracted 41 pulses from the complete 256 pulses to simulate two typical types of SA data, that is, random missing sampling (RMS) and gap missing sampling (GMS) SA data [3]. Figure 4a,b give the range profile sequence for the RMS and GMS, respectively. The background noise is clearly observed because of a low SNR.

**Figure 5:**
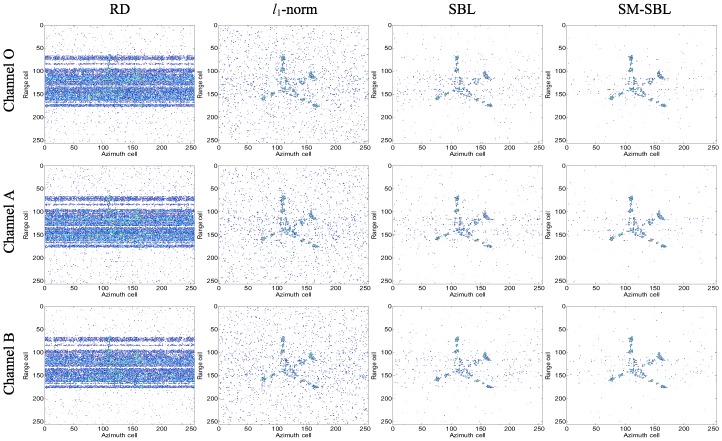
Inverse synthetic aperture radar (ISAR) imagery results for random missing sampling (RMS).

Four ISAR imaging methods, including the RD method, the $l_{1}$ norm-based InISAR imaging algorithm [7], the SBL-based InISAR imaging algorithm [8] and the proposed SM-SBL-based method, are utilized to obtain ISAR images from the RMS and GMS. The imagery results for the RMS are given in Figure 5, in which the first, second, third and fourth columns are the ISAR images obtained by RD, $l_{1}$-norm, SBL and SM-SBL, respectively, and the first, second and third rows give the ISAR images from the channels O, A and B, respectively. It is seen that the results of RD are defocused in this case, which indicate its invalidity for SA data. In contrast, the other three methods obtain well-focused ISAR images, and the images obtained by SM-SBL are relatively less noisy than those obtained by $l_{1}$-norm and SBL, which validates its superior performance in terms of noise suppression against $l_{1}$-norm and SBL. Noting that InISAR imaging requires perfectly matched multi-channel ISAR images for interferometry, the correlation coefficients (CC) of the multi-channel ISAR images were computed to numerically compare the matched degree of ISAR images obtained by different methods. The results are given in Table 1. For purposes of comparison, the CC of the images obtained by RD for the complete data are also given. It is seen that the proposed SM-SBL achieved the largest CC, which validates its superior performance in terms of improving the matched degree of multi-channel ISAR images.

**Figure 6:**
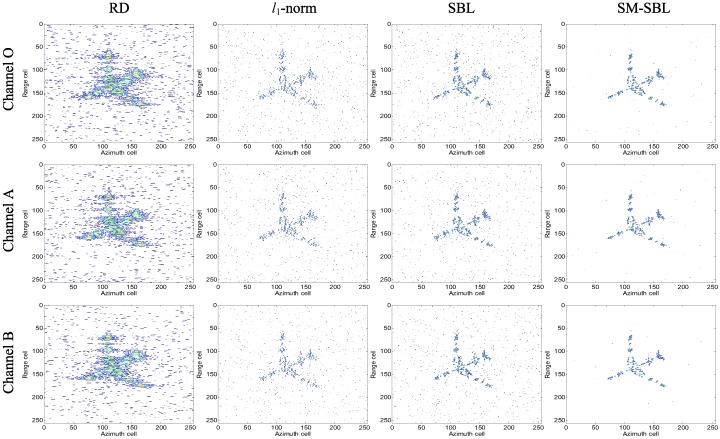
Inverse synthetic aperture radar (ISAR) imagery results for gap missing sampling (GMS).

Similarly, the ISAR images for GMS obtained by the four methods are given in Figure 6, for which each row and column show the ISAR images from the same channel and that are achieved by the same method, respectively. It is seen that RD suffers from high side lobes and strong noise because the pulses for imaging are limited. Again, the other three SSR algorithms successfully obtain high-resolution ISAR images, as shown in the second, third and fourth columns of Figure 6. Compared with $l_{1}$-norm and SBL, the proposed SM-SBL achieves images with a lighter noise floor, which indicates its superior noise suppression performance. Additionally, the CC between ISAR images from different channels achieved by the four methods along with RD for the complete data are given in Table 2, so as to further compare their performance in terms of improving the matched degree of multi-channel ISAR images. It is seen that the proposed SM-SBL obtains the highest CC again, which validates the effectiveness of jointly reconstructing multi-channel ISAR images with SM-SBL. Additionally, it should be noted that RD achieves higher CC than $l_{1}$-norm and SBL, which indicates that RD performs better than SSR for producing matched multi-channel ISAR images. In contrast, although the proposed SM-SBL belongs to SSR, it is still able to obtain higher CC than RD, which further validates its superior performance in terms of improving the matched degree of multi-channel ISAR images.

**Figure 7:**
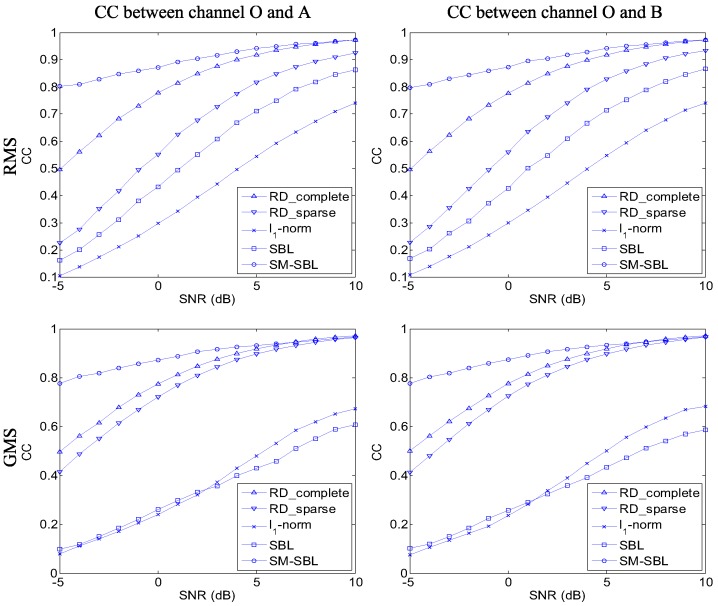
Comparison of correlation coefficients (CC) of multi-channel inverse synthetic aperture radar (ISAR) images under different signal-to-noise ratio (SNR) conditions.

Next, we compare the performances of RD for complete and sparse data, $l_{1}$-norm, SBL and the proposed SM-SBL under different noise conditions. The parameters of this experiment were the same as for the former, except that the SNR ranged from −5 to 10 dB in steps of 1 dB. The curves of CC of multi-channel ISAR images with respect to the SNR obtained by different algorithms are given in Figure 7 to quantitatively compare their performance. It is seen that the proposed SM-SBL obtains the highest CC under any noise levels for both RMS and GMS. For example, the CC obtained by SM-SBL approaches 0.8 for a SNR of −5 dB, while those obtained by RD and SBL are lower than 0.3. Therefore, the proposed SM-SBL performs more robustly than RD, $l_{1}$-norm and SBL in terms of producing well-matched multi-channel ISAR images under low-SNR conditions.

**Figure 8:**
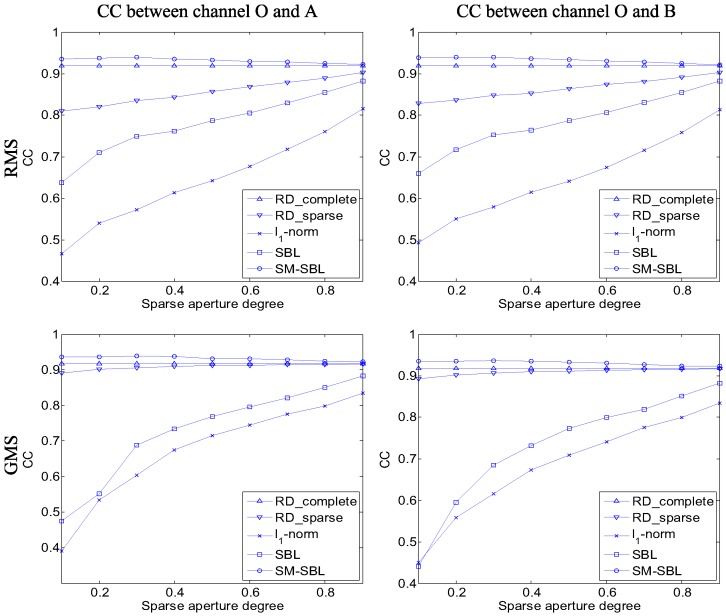
Comparison of correlation coefficients (CC) of multi-channel inverse synthetic aperture radar (ISAR) images under different sparse aperture (SA) degrees.

We next compare the performance of the five methods under different SA degrees. The experimental parameters were the same as for the first experiment, except that the SA degree ranged from 0.1 to 0.9 in steps of 0.1, and the SA degree was defined as the rate of pulses extracted from the complete data. Figure 8 gives the curves of CC with respect to the SA degree obtained by different algorithms. Additionally, the proposed SM-SBL obtains the highest CC under any SA degrees for both RMS and GMS, which further validates its superior performance.

**Figure 9:**
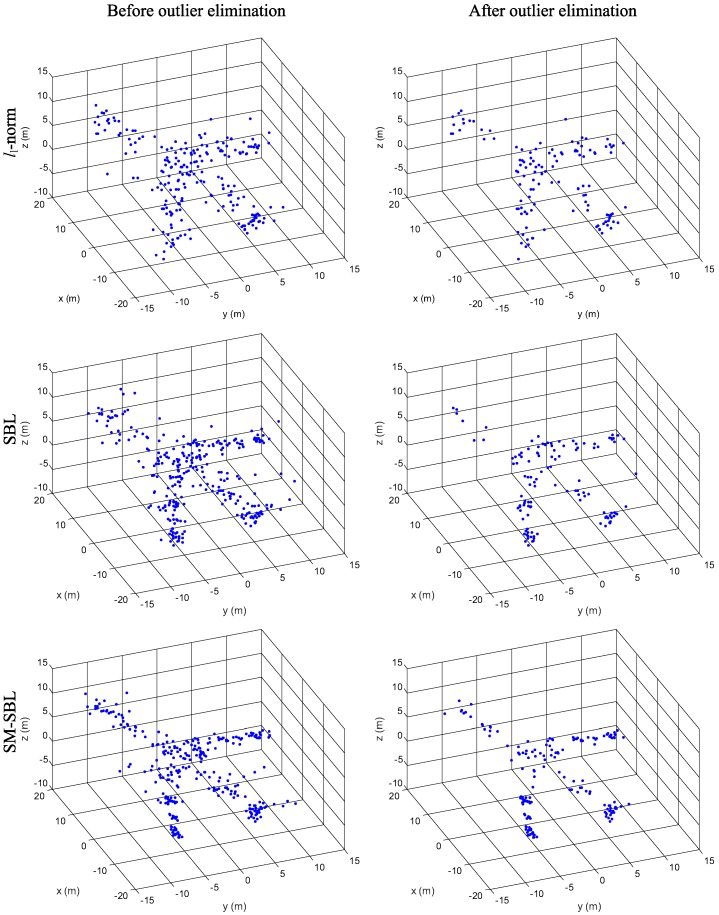
Reconstructed 3-dimensional (3-D) scatterer distributions for random missing sampling (RMS).

**Figure 10:**
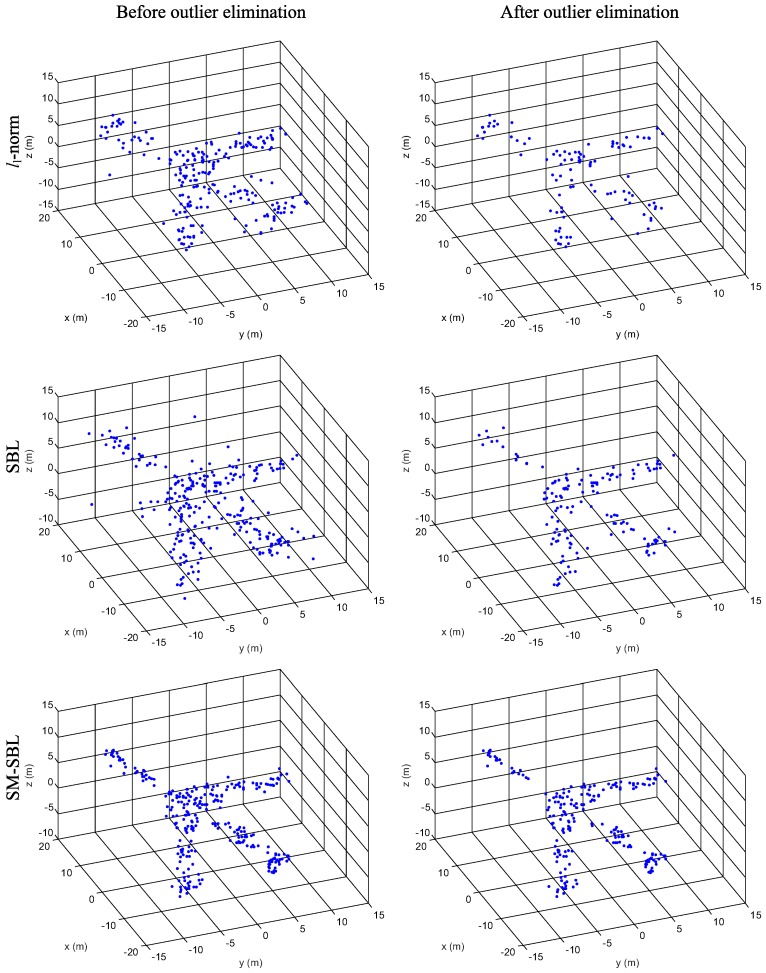
Reconstructed 3-dimensional (3-D) scatterer distribution for gap missing sampling (RMS).

We now compare the 3-D scatterer distributions reconstructed with the multi-channel ISAR images obtained by $l_{1}$-norm, SBL and SM-SBL. The reconstructed 3-D scatterer distributions before and after outlier elimination for RMS are given in Figure 9, for which the first, second and third rows show the results obtained by $l_{1}$-norm SBL and SM-SBL, and the first and second columns give the results before and after outlier elimination, respectively. It is seen that the outliers are successfully eliminated in the 3-D scatterer distributions after outlier elimination, which confirms the effectiveness of the proposed outlier elimination method. Additionally, it is clear that the proposed SM-SBL obtains more realistic 3-D scatterer distributions of airplanes than $l_{1}$-norm and SBL, because it brings improvement in terms of the matched degree of multi-channel ISAR images. Figure 10 gives the 3-D scatterer distributions for GMS. Again, the proposed SM-SBL obtains better results than SBL, which further validates its superior performance. Additionally, it is seen that the 3-D distribution obtained by SM-SBL before outlier elimination is similar to that after outlier elimination, because SM-SBL already achieves satisfactory 3-D distribution with few outliers, and the process of outlier elimination can be omitted. In contrast, the 3-D distributions obtained by $l_{1}$-norm and SBL contain considerable numbers of outliers, which makes outlier elimination a necessity.

Finally, the mean-square errors (MSE) of estimated rotational velocities and computational time of $l_{1}$-norm, SBL and SM-SBL are given in Table 3 to further quantitatively compare their performance. It is seen that the proposed SM-SBL estimates the rotational velocity more accurately than $l_{1}$-norm and SBL. It is also far more computationally efficient because of the sequential update strategy that it utilizes.

## 5. Conclusions

This paper proposes a novel sparse InISAR imaging algorithm. The SM-SBL method is proposed to jointly reconstruct well-matched multi-channel ISAR images with highly computational efficiency. The LSM is utilized to eliminate the outliers and estimate the rotational velocity of the target. On the basis of numerous experimental results, we are confident to draw the conclusion that the proposed sparse InISAR imaging algorithm has two compelling advantages. Firstly, it improves the matched degree of ISAR images from different channels for SA data. For example, when the SNR is −5 dB, the correlation coefficient of multi-channel ISAR images for GMS obtained by the proposed algorithm approaches 0.8, while that obtained by the SBL-based InISAR imaging algorithm is merely 0.4. Additionally, the proposed SA-InISAR imaging algorithm is computationally efficient. Benefitting from the sequential procedure of SM-SBL, the SM-SBL-based SA-InISAR imaging algorithm avoids the time-consuming large matrix inversion. Experimental results indicate the proposed algorithm is 6 times faster than the SBL-based InISAR imaging algorithm.

## Figures and Tables

**Table 1:** Correlation coefficients (CC) of multi-channel inverse synthetic aperture radar (ISAR) images for random missing sampling (RMS).

ISAR Imaging Methods	CC between ISAR Images from Channels O and A	CC between ISAR Images from Channels O and B
Range-Doppler (RD) for complete data	0.9199	0.9204
RD for sparse data	0.8151	0.8289
$l_{1}$-norm	0.5523	0.5422
Sparse Bayesian learning (SBL)	0.7097	0.7048
Sequential multiple sparse Bayesian learning (SM-SBL)	0.9399	0.9384

**Table 2:** Correlation coefficients (CC) of multi-channel inverse synthetic aperture radar (ISAR) images for gap missing sampling (GMS).

ISAR Imaging Methods	CC between ISAR Images from Channels O and A	CC between ISAR Images from Channels O and B
Range-Doppler (RD) for complete data	0.9201	0.9202
RD for sparse data	0.8990	0.9010
$l_{1}$-norm	0.5703	0.5897
Sparse Bayesian learning (SBL)	0.4313	0.4562
Sequential multiple sparse Bayesian learning (SM-SBL)	0.9320	0.9385

**Table 3:** Numerical comparison of algorithm performance.

Algorithm	Mean-Square Errors (MSE)_$\omega_{x}$ for Random Missing Sampling (RMS) (%)	MSE_$\omega_{z}$ for RMS (%)	MSE_$\omega_{x}$ for GMS (%)	MSE_$\omega_{z}$ for GMS (%)	Time (s)
$l_{1}$-norm	12.47	15.14	14.89	16.19	31.3
SBL	8.12	9.79	11.75	6.94	49.5
SM-SBL	4.58	5.02	3.26	4.75	7.3
